# Informed Consent for Surgery at Resumption of Elective Activity After the First Wave of COVID-19

**DOI:** 10.7759/cureus.11642

**Published:** 2020-11-23

**Authors:** Nourelhuda M Darwish, Muhammad Rafaih Iqbal, Adeel Abbas Dhahri, Neville Jacob, Jennifer Jebamani, Amy Easthope, Vardhini Vijay

**Affiliations:** 1 General Surgery, Princess Alexandra Hospital NHS Trust, Harlow, GBR

**Keywords:** covid-19, elective surgery, consent, litigation

## Abstract

Background

The coronavirus disease 2019 (COVID-19) pandemic has changed the dynamics of healthcare, and the elective surgical consent process has also evolved. The Royal College of Surgeons of England published guidance on consent during COVID-19. Through this study, we aimed to assess our local consent adherence to these guidelines on the resumption of elective activity after the first wave of COVID-19.

Methods

This prospective review of consecutive elective surgical consent forms was conducted from 20^ ^July 2020 to 16 August 2020 at the Princess Alexandra Hospital NHS Trust, England. The primary outcome was evidence of COVID-19 risk documentation on the consent forms.

Results

A total of 116 patients’ consent forms were reviewed. Most patients were American Society of Anaesthesiologists (ASA) grade 2 (n=70; 60.34%). Only 25 consent forms (21.55%) had COVID -19 and its associated risks documented, with registrars being the most compliant (19/46; 41.3%) followed by consultants (6/51; 11.7%). With regards to the surgical sub-specialities, general surgery, orthopaedics and ENT had the highest compliance with the guidance.

Conclusions

As the elective activity resumes, peri-operative risks of COVID-19 should be weighted in during the informed consent process, as mentioned in the latest international guidelines on consent to avoid litigation and negligence claims.

## Introduction

The novel coronavirus disease 2019 (COVID-19) pandemic, since its origin in Wuhan, has caused a significant healthcare crisis across the world [1-4]. This led to a shift of primary focus onto emergency care with a significant impact on elective activities across the globe [5]. Approximately 28 million operations were cancelled or postponed globally during the peak of 12 weeks of the pandemic, with 2.3 million cancellations per week [6]. In the United Kingdom, approximately 36,000 cancer surgeries have been cancelled, and clearing this backlog will require a minimum of 11 months with 20% extra activity and would cost approximately £2 billion [6]. Most or almost all of the elective activity was put to halt during the peak of the pandemic owing not only to the overwhelming capacity issues but also to the risk of mortality associated with the peri-operative period.

A mortality of 19% has been reported in patients undergoing elective surgery who were diagnosed with COVID-19 peri-operatively [7]. With the first wave of COVID-19 pandemic finished, hospitals are resuming back the elective activities. This brings in to light the importance of informed consent, especially with COVID-19 being still prevalent in the community and the material risk to life due to its associated complications. Following the landmark case of Montgomery vs Lanarkshire Health Board (2015) UKSC 11, doctors must take reasonable care to ensure that patients are aware of any material risks involved in the recommended treatment and any reasonable alternative treatment [8]. The Royal College of Surgeons of England initially published guidelines for “recovery of surgical services during and after COVID-19”, which was updated in June 2020 with the addition of a new tool called “consent to treatment, while COVID-19 is present in society” [9]. The American Society of Plastic Surgeons published a similar guideline during the same period [10]. An essential and critical component of the new guidelines is that patients must be informed about the risk of COVID-19 infection and related complications.

Failure to undertake informed consent appropriately has led to litigation claims in the past. It has been estimated that approximately 3.4-6 % of all such claims are related to the inadequacy of the informed consent [11]. We undertook this study to look into the adherence to the Royal College of Surgeons of England guidelines related to consent to treatment on the resumption of elective activity at the Princess Alexandra Hospital NHS Trust (PAH).

## Materials and methods

Study design

A prospective review was conducted of all the consecutive patients undergoing elective surgery following the resumption of these services after the first wave of COVID-19 pandemic at PAH.

Time period of data collection

Data were collected over a time period of four weeks (20 July 2020 to 16 August 2020).

Setting

PAH serves a population of about 500,000 primarily in the West Essex and East Hertfordshire, England. The elective activity was halted at our trust during the first wave of COVID-19, and all urgent non-emergency operating was done at a local private hospital which was a dedicated cold/green site. On the resumption of elective activity, all these patients were swab tested for severe acute respiratory syndrome coronavirus-2 (SARS-CoV-2) pre-operatively and were sent an information leaflet related to the risk of COVID-19. All the consent forms were reviewed in the immediate post-operative period, and data were entered on a pre-designed data collection form.

Outcome

The primary outcome was evidence of COVID-19 risk documentation on the consent form.

Data collection

Hospital electronic records and patient notes were reviewed. Data were collected regarding patient demographics, co-morbidities, pre-operative COVID-19 status, speciality, the grade of the surgeon consenting, documentation of COVID-19 risk and its related complications on the consent form, operation details, length of stay, post-operative COVID-19 testing, 30-day readmission, complications and mortality. Operation complexity was classified into minor, intermediate and major/complex as per the NICE guidelines [12]. Complications were classified as per the Clavien-Dindo classification [13].

Data analysis

Categorical variables were calculated as number and percentage, while continuous variables were calculated as median and interquartile range.

Ethical consideration

This study was exempted from ethical approval and was registered as an audit with the local audit department.

## Results

A total of 116 patients were included in the study, with a median age of 70 years (interquartile range [IQR]: 58-80 years). Of the 116 patients, 63 (54.3%) were males, while 73 (62.9%) had a body mass index (BMI) less than 30. Thirteen (11.2%) patients were smokers. Most (n= 70; 60.34%) of the patients were American Society of Anaesthesiologists (ASA) grade 2, 22 (18.96%) were ASA grade 3, and 19 (16.37%) were ASA grade 1 (Table 1).

**Table 1:** : Baseline patient demographics n = 116 ASA, American Society of Anaesthesiologists; BMI, body mass index; IQR, interquartile range

Variable	Number (%)
Median age, years	70 (IQR: 58–80)
Gender	
Male	63 (54.3)
Female	53 (45.6)
BMI	
<30	73 (62.9)
>30	43 (37.0)
Current smoker	13 (11.2)
Co-morbidities	
Diabetes mellitus	13 (11.2)
Ischemic heart disease	14 (12)
Hypertension	49 (42.2)
Asthma	11 (9.8)
Chronic obstructive lung disease	9 (7.75)
ASA grade	
ASA 1	19 (16.37)
ASA 2	70 (60.34)
ASA 3	22 (18.96)
ASA 4	5 (4.31)

Most (n= 72; 62.06%) of the operations were classified as minor followed by intermediate (n=28; 24.14%) (Table 2).

**Table 2:** Preoperative, operative and post-operative data n = 116 ENT, ear, nose and throat; LOS, length of stay

Variable	Number n (%)
Pre-Operative	
Speciality	
General surgery	24 (20.6)
Urology	18 (15.5)
ENT	15 (12.9)
Gynaecology	12 (10.3)
Maxillofacial surgery	20 (17.3)
Ophthalmology	13 (11.2)
Orthopaedics	4 (3.4)
Oral surgery	10 (8.6)
Operative	
Anaesthesia	
General anaesthetic	69 (59.5)
Local anaesthetic	46 (39.6)
Spinal anaesthetic	1 (0.86)
Operation classification	
Minor	72 (62.06)
Intermediate	28 (24.14)
Major or complex	16 (13.79)
Post-Operative	
LOS	
Day case	105 (90.5)
1 day	4 (3.4)
3 days	2 (1.7)
8 days	1 (0.86)
12 days	1 (0.86)
16 days	1 (0.86)

All patients had had negative COVID swabs pre-operatively. Of the patients, 47 (40.5%) underwent surgery using a local anaesthetic, of which 89.3% (42/47) had no documentation of COVID-19 risk on the consent forms (Table 3).

**Table 3:** COVID-19 risk documentation on the basis of type of anaesthetic

Anaesthetic	COVID-19 risk documented on consent form	COVID-19 risk not documented on consent form
General anaesthesia	19 (27.5%)	50 (72.5%)
Local anaesthesia	05 (10.6%)	42 (89.4%)

Looking at the different surgical sub-specialities, general surgery, orthopaedics, ENT (ear, nose and throat) and urology were compliant with the guidelines (Table 4).

**Table 4:** Compliance to the Royal College of Surgeons of England guidelines on the basis of sub-specialities ENT, ear, nose and throat

Speciality	COVID-19 risk documented on consent form	COVID-19 risk not documented on consent form
General surgery (n = 24)	13 (54.17%)	11 (45.83%)
Orthopaedics (n = 4)	3 (75%)	1 (25%)
Urology (n = 18)	1 (5.56%)	17 (94.44%)
ENT (n = 15)	8 (53.33%)	7 (46.67%)
Gynaecology (n = 12)	0 (0.0%)	12 (100%)
Maxillofacial (n = 20)	0 (0.0%)	20 (100%)
Oral surgery (n = 10)	0 (0.0%)	10 (100%)
Ophthalmology (n = 13)	0 (0.0%)	13 (100%)

Only 25 (21.55%) patients had COVID -19 and its related complications documented in their consent forms, with registrars (19/46; 41.3%) being the most compliant followed by consultants (6/51; 11.7%). None of the patients consented by senior house officers (0/17; 0%) or associate specialists (0/2) had the risk of COVID-19 infection documented on the consent forms (Table 5).

**Table 5:** COVID-19 risk documentation on consent forms

Variable	n (%)	Grade of person consenting, n (%)			
		Consultant, n = 51	Associate specialist, n = 2	Registrar, n = 46	Senior house officer, n = 17
COVID-19 risk documented on consent form	25 (21.55)	6 (11.7)	0 (0.0)	19 (41.3)	0 (0.0)
COVID-19 risk not documented on consent form	91 (78.4)	45 (88.2)	2 (100)	27 (58.69)	17 (100)

In the 30-day follow-up period, three (2.5%) patients were re-admitted. Out of these readmissions, one was for a chest infection (non-COVID-19 related), one for exacerbation of congestive cardiac failure and one for bile leak for laparoscopic cholecystectomy. Five (4.3%) patients developed complications in the 30-day follow-up period. There was no 30-day mortality in this study (Table 6).

**Table 6:** Thirty-day follow-up

	Number (%)
Mortality	
COVID-19 related	0 (0)
Non-COVID-19 related	0 (0)
Readmission	3 (2.5)
Complications	5 (4.3)
Clavien-Dindo complication and grade	
Grade 1 (post-operative hematuria)	1 (0.86)
Grade 2 (wound infection, high stoma output and paralytic ileus with acute cholecystitis)	3 (2.5)
Grade 3a	0 (0)
Grade 3b (bile leak)	1 (0.86)
Grade 4a	0 (0)
Grade 4b	0 (0)
Grade 5	0 (0)

## Discussion

This study highlights the importance of documentation of risks, especially COVID-19 related risks during the informed consent process in addition to the usual surgical and anaesthetic risks.

Informed consent is a core component of any surgical procedure, whereby the surgeon informs the patient about the available options for treatment and help the patient to make an informed decision [14]. COVID-19 has made the informed consent more critical and crucial than ever as the knowledge and understanding of the effects of COVID-19 on post-operative patient outcomes is limited, and the possibility of acquiring COVID-19 while in the hospital environment is present.

The first study on the perioperative outcomes in patients undergoing elective surgery reported a 100% incidence of post-operative pneumonia, with one-third of them developing acute respiratory distress syndrome (ARDS) with an overall mortality of 21% [15]. Another study reported an overall 30-day mortality of 19.1% due to COVID-19 after elective surgery in the perioperative period [7]. Tests for COVID-19 that are currently available have a varying degree of false-negative rates. The median false-negative rate on the day of onset of symptoms has been reported to be 38% decreasing to 20% on day 8 [16]. This raises the possibility of patients with false-negative results and asymptomatic undergoing elective surgery, which obviously increases the risk of development of perioperative COVID-19 related complications. With these issues in mind, the importance of informed consent is further highlighted.

In our study, only 21.5% of the patients had documentation of COVID-19 related risk on their consent form. The latest Royal College of Surgeons of England guidance on consent clearly states that additional considerations should be discussed with the patient as a part of the informed consent process [9]. These include the following:

1. The risk of contracting COVID-19 while in the hospital.

2. The risk of operation for patients who have tested positive for COVID-19.

3. Changes in the coordination of care due to pandemic response and a possibility of the scarcity of the resource.

Whilst it is not possible to give an accurate estimate of the risk of contracting COVID-19 while the patient is in the hospital, even then as such material risk is present and therefore it should be discussed with the patient as a part of informed consent. Different international studies have mentioned higher risk associated if a patient has a close contact with a COVID-19 positive person while in the hospital [17,18]. This is a similar risk to hospital-acquired infection before the pandemic. Not informing patients of such risk put the clinician susceptible to clinical negligence claims. In line with the 2015 decision of the Supreme Court in the case of Montgomery vs Lanarkshire Health Board, doctors have a duty to warn a patient of a material risk inherent in the proposed treatment and discuss this with them [8,19].

## Conclusions

The findings of this study suggest that only 21.5% of the patients were consented for risks of acquiring COVID-19 and its related complications. As the elective activity resumes, peri-operative risks of COVID-19 should be weighted in during the informed consent process, as mentioned in the Royal College of Surgeons of England guidelines on consent to avoid litigation and negligence claims.
